# Low cost and scalable method for modifying surfaces of hollow particles from hydrophilic to hydrophobic†

**DOI:** 10.1039/d0ra06114j

**Published:** 2020-08-21

**Authors:** Jaswinder Sharma, Georgios Polizos, Diana Hun, Kashif Nawaz, Ritu Sahore

**Affiliations:** Roll-to-Roll Manufacturing Group, Energy and Transportation Science Division, Oak Ridge National Laboratory Oak Ridge TN 37831 USA sharmajk@ornl.gov +1-865-241-2333; Building Technologies Research & Integration Center, Oak Ridge National Laboratory Oak Ridge TN 37831 USA

## Abstract

Hydrophobic hollow silica particles are desirable for several applications such as hydrophobic coatings, thermal insulation, and thermally resistant insulative paints. However, converting hydrophilic particles into hydrophobic particles without compromising their structural integrity is challenging. In this work, we present a low cost strategy to modify the surface of hollow silica particles from hydrophilic to hydrophobic without compromising their structural integrity.

## Abstract 2

A low cost strategy is developed for hydrophobization of hollow silica particles without compromising with their structural integrity.

## Graphical abstract

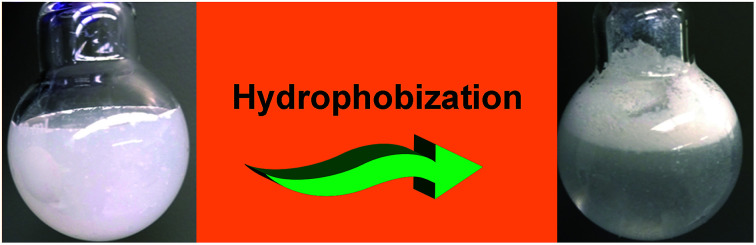

## Introduction

Hollow silica particles are a unique class of materials that has applications in various fields such as drug delivery, thermal insulation, supercapacitors, battery electrolytes, phase change materials, and superhydrophobic coatings.^1–14^ There are several strategies for synthesizing hollow silica particles such as use of polymer templates, polymer micelles, spray drying, ultrasonic spray pyrolysis, viruses, bacteria, and solid silica particles.^15–21^ Although the synthesis of hydrophilic silica particles is well developed, several applications require hydrophobic hollow silica particles.^8,9^ For such applications, a hydrophobic silane coating is typically added to the outer surface of the hollow silica particle shell using solution phase strategies.^22–24^ For example, trimethoxymethylsilane or triethoxyethylsilane are added to either alcohol or toluene solutions with hollow silica particles while stirring. Although these solution-phase strategies are suitable for solid silica particles, they are difficult to apply to hollow silica particles because solution-phase coating of hydrophobic silanes requires mixing and stirring for several hours. Solid particles can withstand this process, however, we noticed that a fraction of hollow silica particles breaks (Fig. 3) during this process because of fragile nature of silica shell. The broken particles can deteriorate some of their desired properties, for example, can enhance the thermal conductivity and thus reduce their attractiveness as thermal insulation material.

Additionally, solution-phase grafting of hydrophobic silanes is an expensive process because it wastes large amounts of solvents (*e.g.*, alcohol and toluene). Therefore, there is a need for an approach for making a hydrophobic silane coating on hollow silica particles without compromising their shell integrity while keeping the costs lower.

Herein, we developed a strategy to modify the surface of hollow silica particles using vapors of hydrophobic silane that is economical and does not compromise the structural integrity of the particles. We also identified the silanes that can be used in vapor-phase coatings.

Though, a similar technique, chemical vapor deposition (CVD) of silanes has been investigated before for making thin films on flat surfaces,^25,26^ however, CVD needs expensive instrumentation and very low vacuum, and can only be used for flat surfaces. Advantages that our approach offers over CVD of silanes^25^ include that it does not need expensive instrumentation (*e.g.*, CVD chamber) and very low vacuum, and is applicable to both flat surfaces and particle powders. This approach is the first vapor-based approach to modify the surface of hollow silica particles from hydrophilic to hydrophobic.

## Experimental

### Chemicals

Tetraethyl orthosilicate (TEOS), trimethoxymethylsilane (TMMS), triethoxymethylsilane (TEMS), trimethoxyoctylsilane (TMOS), ammonium hydroxide (28–30% of NH_3_ in 100 mL of water), and styrene were purchased from Sigma Aldrich. 2,2′-Azobis(2-methylpropionamidine)dihydrochloride was purchased from ACROS-Organics – Fisher Scientific. Isopropanol was purchased from Fisher Scientific.

### Polystyrene particle synthesis

Styrene (4.54 g) was added to the water (200 mL). The reaction mixture was allowed to heat for 30 minutes at 68 °C. Afterward, 2,2′-azobis(2-methylpropionamidine)dihydrochloride (320 mg) was added to this stirring (500 rpm) reaction mixture, and the reaction continued for approximately 12 hours. The final concentration of styrene and 2,2′-azobis(2-methylpropionamidine)dihydrochloride was 0.21 M and 5.8 mM, respectively. Monodisperse particles approximately of 315 nm in diameter were obtained. Fig. S1 (ESI†) shows the scanning electron microscope (SEM) images of polystyrene particles.

### Hollow silica particle synthesis

0.5 g of polystyrene particles were mixed in an isopropanol/water (160 cm^3^ isopropanol, 40 cm^3^ water) solution. 2 mmol of tetraethyl orthosilicate was added to make shells around the polystyrene cores. Ammonium hydroxide solution (28–30%; 28–30 g NH_3_ in 100 mL water, from Sigma Aldrich) was used as a catalyst. The core–shell particles were heated at 550 °C for 4 hours to remove the polystyrene core. To scale up the production of hollow particles, the synthesis was proportionately increased. Fig. S2 (ESI†) shows the SEM images of the obtained hollow silica particles.

### Particle characterization

Scanning Electron Microscopy (SEM) imaging was performed by using a Merlin 200 microscope. Both polystyrene and hollow particles samples were deposited on a silicon wafer, which was in turn attached to the SEM stubs by using carbon tape, before performing the SEM imaging. Carbon tape was used in order to minimize the charging on particle surface.

### Formation of hydrophobic coating

**Fig. 1:**
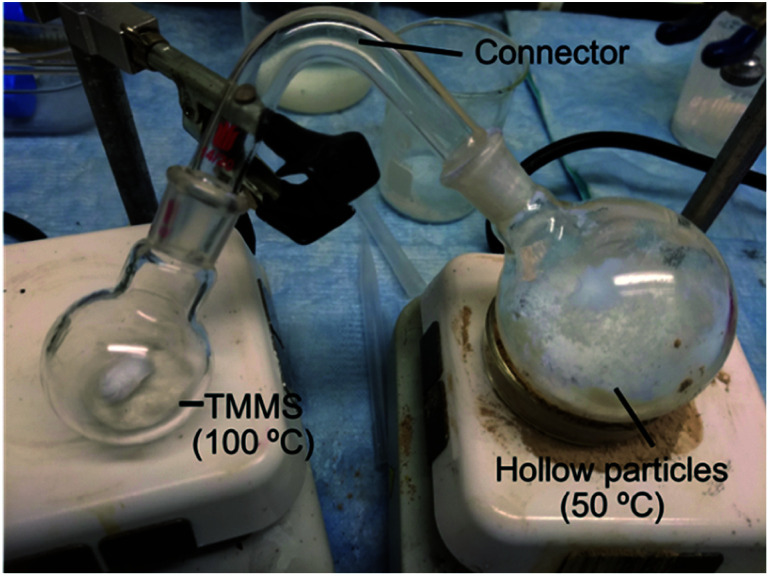
Setup used to make a vapor-phase coating on hollow silica particles.

Fig. 1 shows the setup used to make the hydrophobic coating. We used two round bottomed flasks that were connected through a glass connector. One flask contained 1.0 cm^3^ of hydrophobic silane and the other 15 cm^3^ of hollow silica particles. The flask with the hydrophobic silane was heated to 100 °C to vaporize the silane. The flask with the hollow particles was heated to ≈50 °C to enhance the conjugation process. We shook the samples every 10 minutes to make sure that most of the particles were coated. Other similar setups can be used for vapor formation, deposition, and shaking.

### Measurement of thermal conductivity

Thermal conductivity measurements were performed using a Transient Plane Source (TPS 2500 S) instrument with manufacturer provided sample holder and sensor C5465. All measurements were done on loose particle powders without additional compacting.

### Measurement of moisture adsorption

Moisture absorption/desorption experiments were performed using a dual vapor gravimetric sorption analyzer. Samples were dried overnight at 50 °C and placed in the measurement chamber of the instrument. The moisture adsorption and desorption isotherms were generated at room temperature conditions (25 °C and 1.0 atm). The initial mass of the samples was about 2.0 mg. Each data point (Fig. 5) represents a steady state moisture adsorption capacity at a specific relative humidity. The steady state behavior was established by ensuring that the mass of the sample did not change more than 0.001% between two consecutive readings that were taken 1.0 minute apart. The duration of each step varied based on the time required to reach steady state.

## Results and discussion

We tested three silanes: triethoxymethylsilane (TEMS), trimethoxymethylsilane (TMMS), and trimethoxyoctylsilane (TMOS). TEMS and TMOS didn't provide any coating. Table 1 shows the outcome of different silanes used.

**Table 1:** Silanes that were tested to make the hydrophobic coating along with their chemical formulas. Bold text shows the hydrophobic functional group in each silane

Name	Chemical formula	Coating formed (Yes/No)
TEMS	**H_3_C**–Si(OC_2_H_5_)_3_	No
TMMS	**H_3_C**–Si(OCH_3_)_3_	Yes
TMOS	**H_17_C_8_**–Si(OCH_3_)_3_	No

**Fig. 2:**
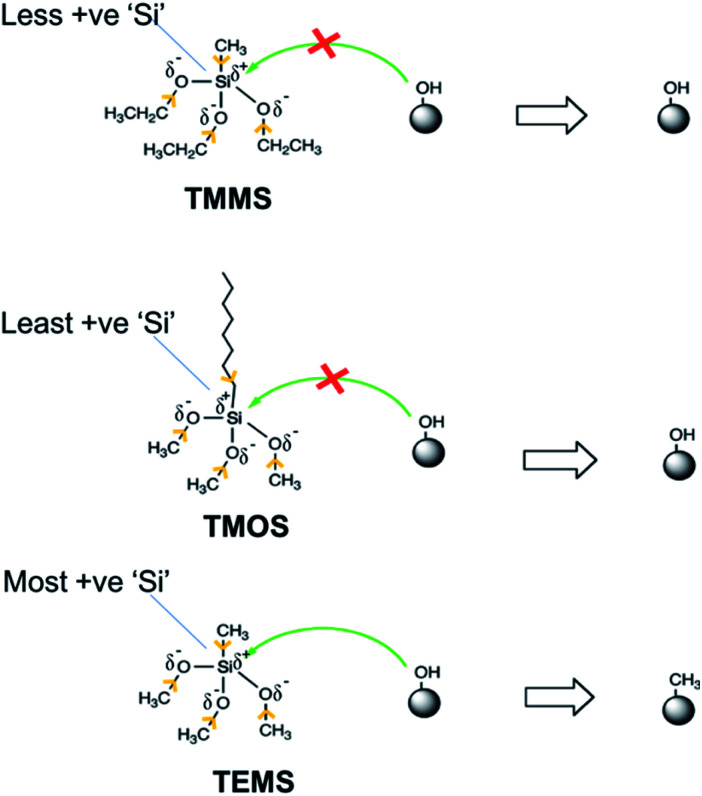
Schematic demonstrating the electron donating inductive effect of alkoxy and alkyl groups on the ‘partial +ve charge’ on the silicon atom, and possibility of different silanes' conjugation to the particle surface.

The inability of TEMS to produce a hydrophobic coating results from its higher stability, which makes it difficult for –OH groups on hollow particles to conjugate with the TEMS molecules. Because leaving anion CH_3_CH_2_O^−^ in TEMS has higher −ve charge on ‘O’ due to electron donating inductive effect of –CH_2_CH_3_. The higher −ve charge on ‘O’ makes the anion unstable and thus slows the conjugation with ‘–OH’ groups on particle surface. Additionally, the higher −ve charge on the ‘O’ atom in CH_3_CH_2_O–Si, makes the ‘Si’ atom less electronegative, *i.e.*, less positively charged.^27^ This lowered electronegativity makes it more difficult for the nucleophilic attack of ‘–OH’ groups attached to hollow particles surface on the ‘Si’ atom. Thus, lowered electronegativity of ‘Si’ and instability of leaving anion CH_3_CH_2_O^−^ makes the TEMS unsuitable for our vapor-phase coating approach. Similarly, in case of TMOS, the increased electron donating inductive effect of the octyl chain makes the ‘Si’ atom less electronegative and thus difficult to attach to the –OH groups of the hollow silica particles, making TMOS also unsuitable for our purposes. Because of the lesser electron donating inductive effect of the –CH_3_ group, the negative charge on the ‘O’ atom in the CH_3_O^−^ anion is lesser than its counterpart (CH_3_CH_2_O^−^) in TEMS and TMOS, which makes it a stable leaving group compared to CH_3_CH_2_O^−^. Similarly, the lesser electron donating inductive effect of the –CH_3_ group directly attached to the ‘Si’ atom and lesser −ve charge on ‘O’ atom of CH_3_O^−^ anion, makes the ‘Si’ atom more electronegative and more susceptible to nucleophilic attack of –OH groups attached to particle surface, thus making the conjugation process possible under normal conditions.^28^Fig. 2 shows the schematic of possible inductive effects of different groups and their effects on the conjugation with –OH groups on hollow silica particles. Yellow arrows indicate the electron donating nature of different functional groups (more details in ESI-S1†).

We observed that conjugation of hydrophobic silane was incomplete when the as synthesized hollow silica particles were used. This occurred because heat treatment at 550 °C resulted in removal of most of the –OH groups from the surface of the particles, which indirectly decreases the number of silane molecules that can attach to the particles' surfaces. In contrast, leaving the particles overnight at ambient conditions results in regeneration of –OH groups on the surface of the particles, which leads to efficient conjugation of TMMS molecules on the surfaces of the particles.

**Fig. 3:**
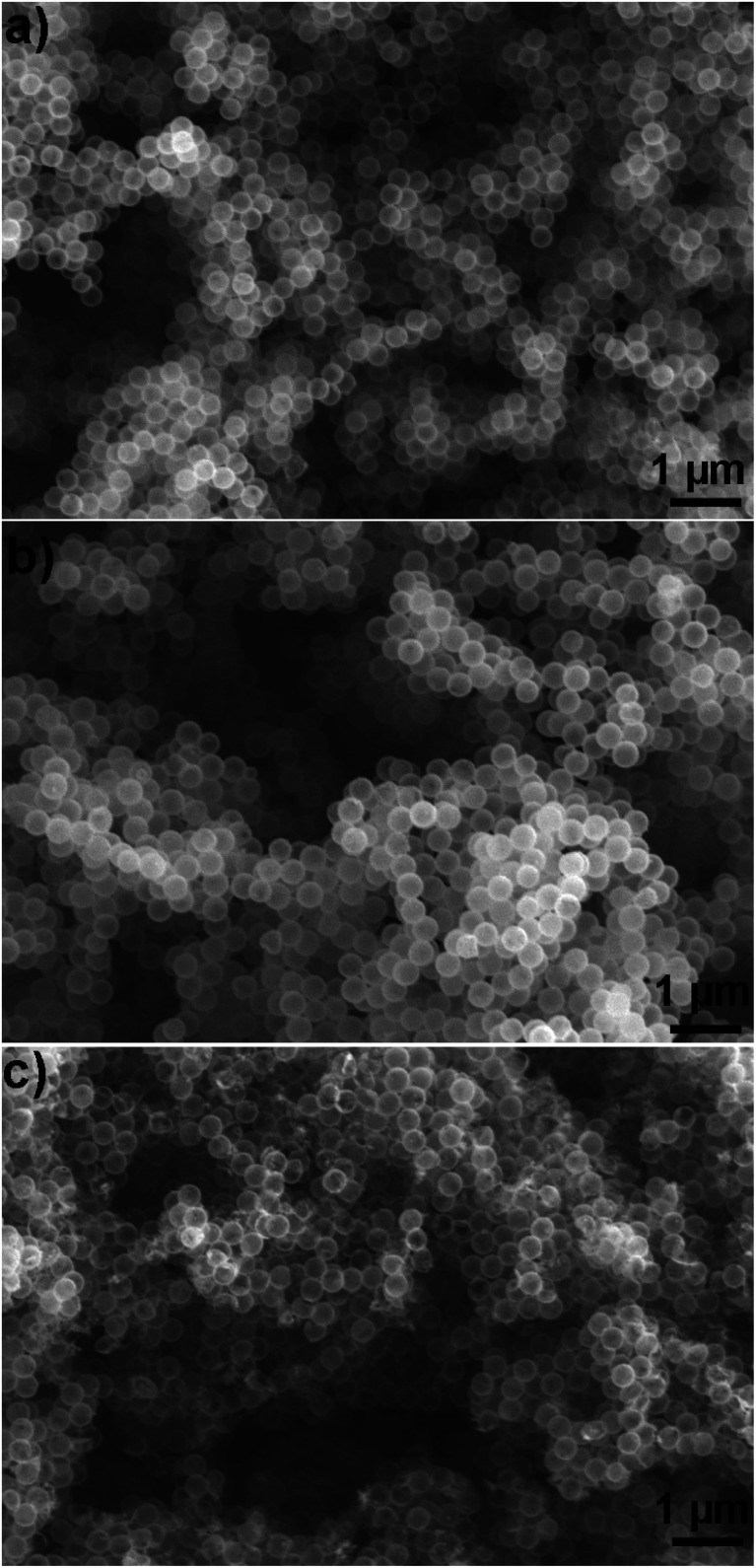
Effect on particle structural integrity of coating method. (a) Pristine particle, (b) coated by using our vapor phase approach, and (c) coated by using solution phase method.

To investigate if the particles retained their structural integrity after coating with hydrophobic silanes, we gathered SEM images that showed that both uncoated (Fig. 3a) and coated (Fig. 3b) particles were similar. In contrast, SEM images (Fig. 3c) of particles coated with the solution-phase approach (experimental details in S1-ESI†) showed a fraction of broken particles.

Therefore, we conclude that our vapor-based approach is superior than the solution-based approach in preserving the structural integrity of the hollow silica particles.

**Fig. 4:**
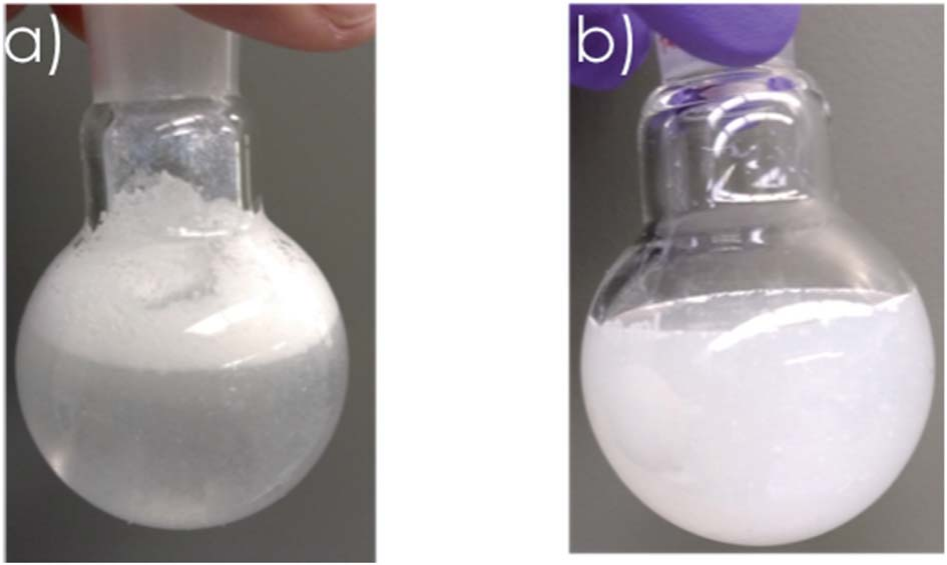
Mixing behaviour of coated and uncoated hollow particles. (a) coated particles floated on water for at least a month, (b) uncoated particles right after mixing and shaking.

To investigate the effectiveness of our vapor-based hydrophobization, we added coated and uncoated particles to flasks with water. Coated particles stayed on the surface of water for at least a month (Fig. 4a), because hydrophobic methyl groups on particles surfaces did not let water to wet the particles. In contrast, uncoated particles, which mostly had –OH groups on their surfaces, mixed quickly with water (Fig. 4b).

To further investigate the formation of hydrophobic silane deposition, we performed infrared spectroscopy studies on uncoated and coated samples. Infrared spectra of coated particles showed a subtle difference from uncoated particles (Fig. S3, ESI†). A decrease in intensity of ‘Si–O–Si’ symmetric stretching (1082 cm^−1^) with missing shoulder, and other Si–O–Si peaks at 778 cm^−1^ and 475 cm^−1^ was observed. Similar to the previous studies,^29^ we also couldn't see a distinct peak of –CH_3_ group because of very low concentration of –CH_3_ groups compared to ‘Si–O–Si’ network.

**Fig. 5:**
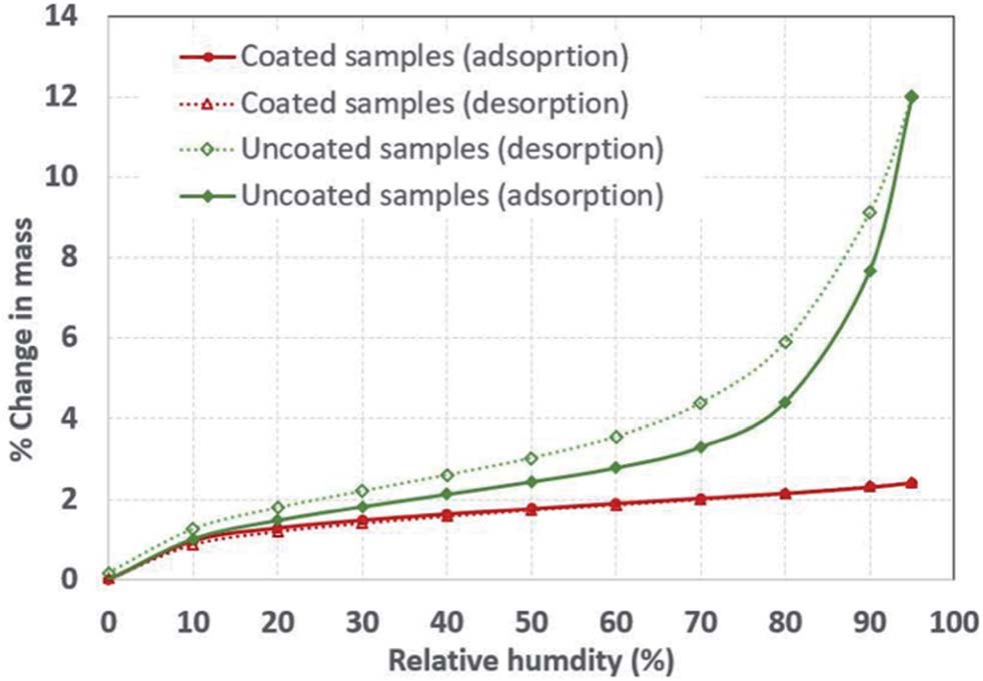
Adsorption and desorption of water vapor at different relative humidity levels of uncoated particles and particles that were coated with trimethoxymethylsilane.

To evaluate the effectiveness of the hydrophobic silane coating, we subjected treated and untreated hydrophobic silane particles to different relative humidity (RH) conditions and evaluated the amount of adsorbed water vapors. Fig. 5 shows the change in mass (amount of moisture adsorbed and desorbed) of the hollow particles at different ‘RH’ levels. The plot indicates that the amount of water vapors adsorbed by the uncoated particles increased linearly with relative humidity until about 70% RH; afterwards, the change in mass showed an exponential growth with ‘RH’. On the contrary, the coated particles displayed minimal adsorption of water vapors regardless of ‘RH’, which indicates the effectiveness of the hydrophobic coating in repelling water vapors.

Additionally, we measured the water contact angle^30,31^ of glass slides coated with hydrophobic silane modified hollow particles and unmodified hollow particles. Glass slide with hydrophobic particles showed a water contact angle of 147.6° ± 2.7°, while glass slide coated with unmodified particles had a contact angle of 25.6–3.1° (experimental details in ESI-S2†). We also measured the Young's modulus of hollow silica particles, which was ≈ 1.5 ± 0.5 GPa (experimental details in ESI-S2†).

We also measured the thermal conductivity of coated and uncoated particles. The coated particles and uncoated particles showed almost similar thermal conductivity (23 ± 1 mW m^−1^ K^−1^). We assume, almost no effect on thermal conductivity arises from very thin nature of coating (monolayer of TMMS molecules). Therefore, our coating process does not interfere with the required qualities such as thermal conductivity and structural stability while it lowers the moisture adsorption by particles.

## Conclusions

We demonstrated a vapor phase strategy to convert hydrophilic hollow silica particles to hydrophobic hollow silica particles. This strategy is especially useful to hollow particles with thin shells (<20 nm) that are prone to structural disintegration when coated using solution-phase processes. Additionally, this approach is cost effective since it does not include wastage of solvents. The process can be used for large scale and continuous processes in which silane vapors are introduced into a chamber having material to be coated continually. We anticipate that this study will entice the research community for vapor phase deposition of different coatings on materials that are not suitable for solution-phase coatings.

## Conflicts of interest

There are no conflicts to declare.

## Supplementary Material

RA-010-D0RA06114J-s001
