# Supplementary material for: Low cost and scalable method for modifying surfaces of hollow particles from hydrophilic to hydrophobic
Source: RSC Adv. 2020 Aug 21;10(52):31065–9. doi: 10.1039/d0ra06114j (PMC9056372; doi:10.1039/d0ra06114j)
Supplement: RA-010-D0RA06114J-s001 [file RA-010-D0RA06114J-s001.pdf]

Electronic Supplementary Material (ESI) for RSC Advances.
This journal is © The Royal Society of Chemistry 2020

Electronic Supplementary Information

Low cost and scalable method for modifying surfaces of hollow particles from hydrophilic to hydrophobic

Jaswinder sharma, Gerogios Polizos, Diana Hun, Kashif Nawaz, and Ritu Sahore

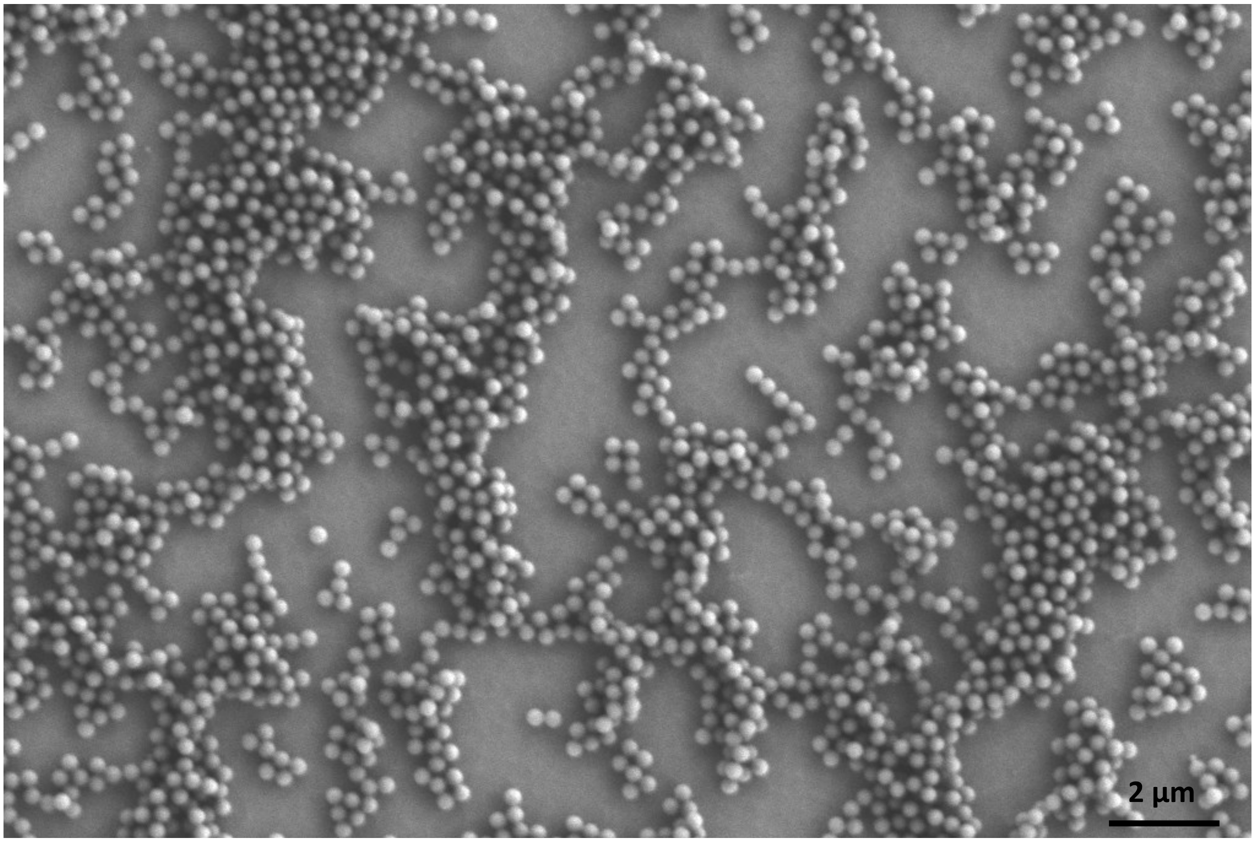

Fig. S1. Polystyrene particles with a dimeter of ≈ 315 nm.

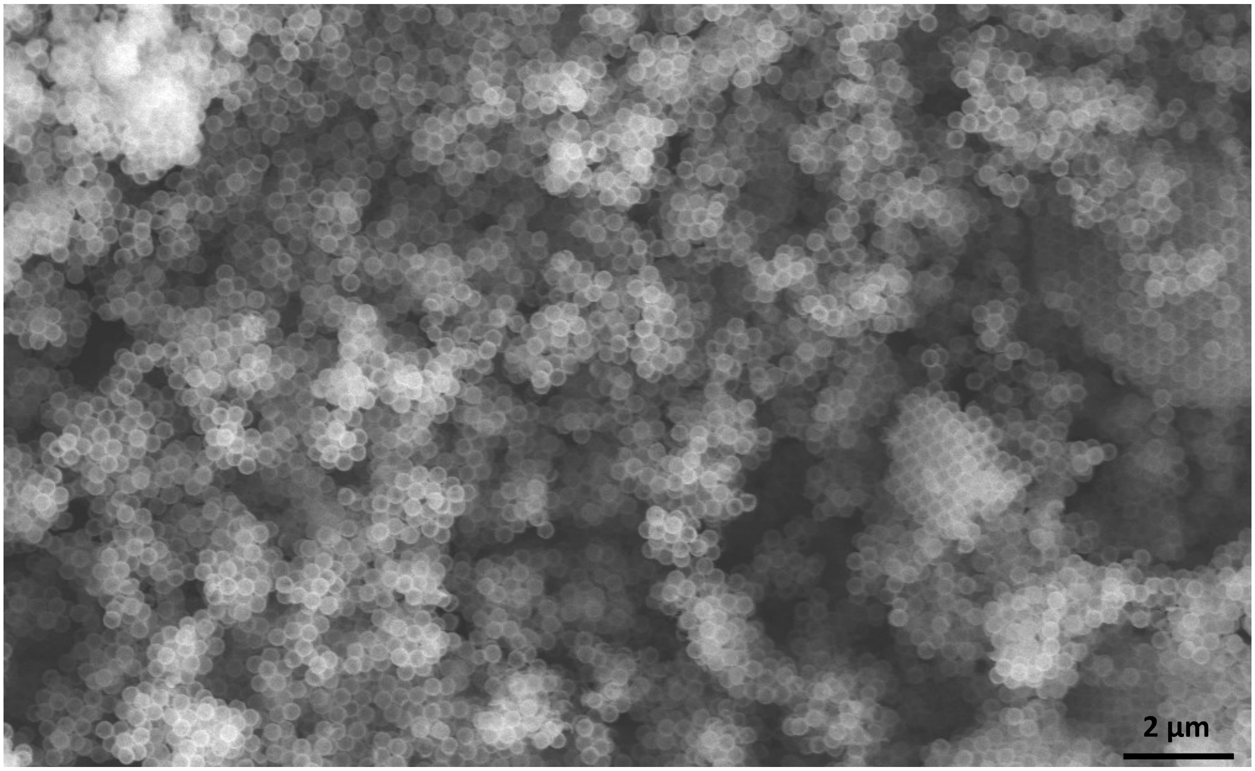

Fig. S2. SEM image of hollow silica particles.

S1.

For SEM imaging of different samples:

Uncoated particles: Dry particles were put on silicon wafer for imaging.

Vapor coated particles: Dry particles were put on silicon wafer for imaging.

Solution phase coating of particles: Pristine hollow silica particles were mixed in toluene and stirred for 15 min. Then TMMS was added to this stirring solution and let the particles stir for 6 hours.

As coated particles were deposited on silicon wafer for SEM imaging.

More on reaction Mechanism:

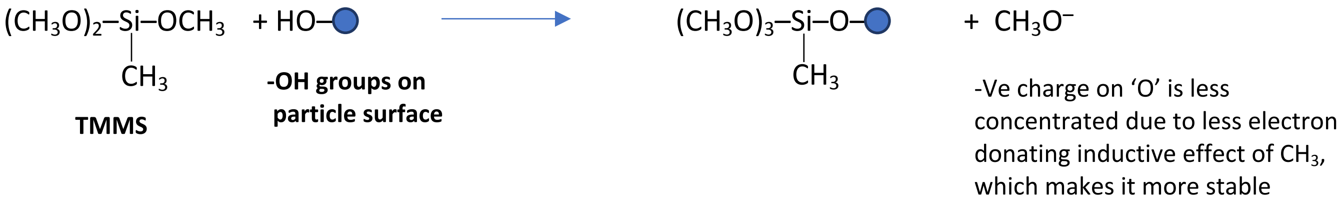

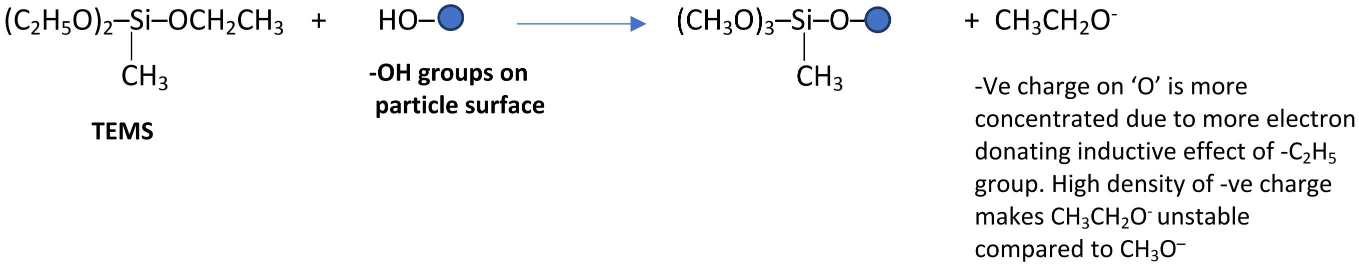

In chemical reactions, if leaving group is less stable, it makes the forward reaction less favorable.

Note: electron donating inductive effect increases with an increase in number of carbon atoms in alky chain, therefore: electron donating inductive effect order is: *Octyl* (C_8H_{17}) *> ethyl* (C_2H_5) *> methyl* (CH_3).

Similarly, as explained in main text, different electron donating effect of different alkyl groups affects differently the partial +ve charge on "Si' atom. Higher the partial +ve charge on "Si' atom, the higher the probability of nucleophilic attack of -OH group attached to particle surface on this "Si' atom, and more favorable the conjugation reaction will be. Similarly, lower the partial +ve charge on "Si' atom, the lower the probability of nucleophilic attack of -OH group attached to particle surface on this "Si' atom, and less favorable the conjugation reaction will be.

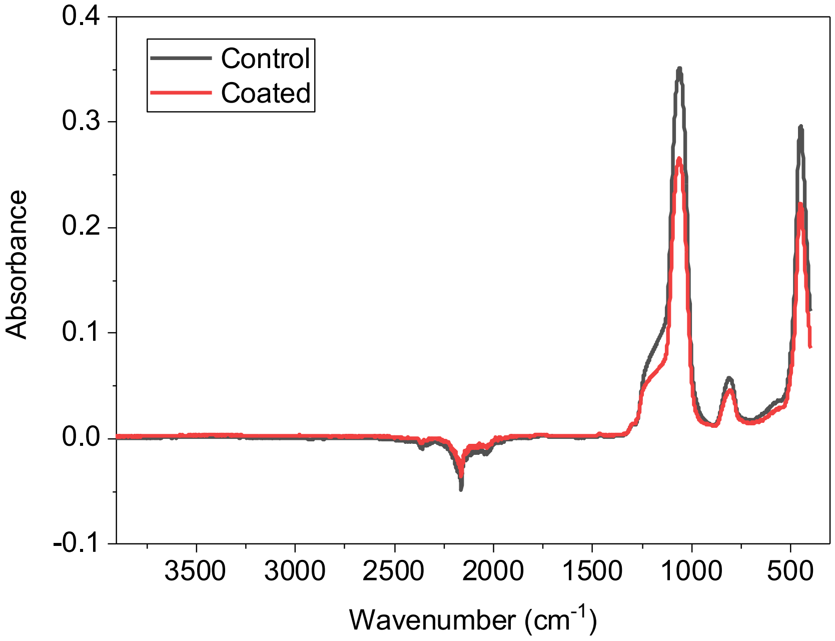

Fig. S3 Infrared spectra of coated and uncoated particles.

S2.

Contact angle measurements:

Water contact angle measurements were performed on an Attension tensiometer (KSV Instruments). Representative images of the sessile drops (5 µL in volume) on the surface of glass slides coated with a thin layer of unmodified and hydrophobic silane modified hollow silica particles are shown in Figure S4. Slides were coated by drop casting a solution of modified particles (in toluene), and unmodified particles (in ethanol). Before measurements, slides were dried at room temperature. The water contact angle on the surface of a glass slide coated with unmodified hollow silica particles was 25.6^{o} ± 3.1^{o}. The water contact angle on the surface of a glass slide coated with hydrophobic silane modified hollow silica particles was 147.6^{o} ± 2.7^{o}.

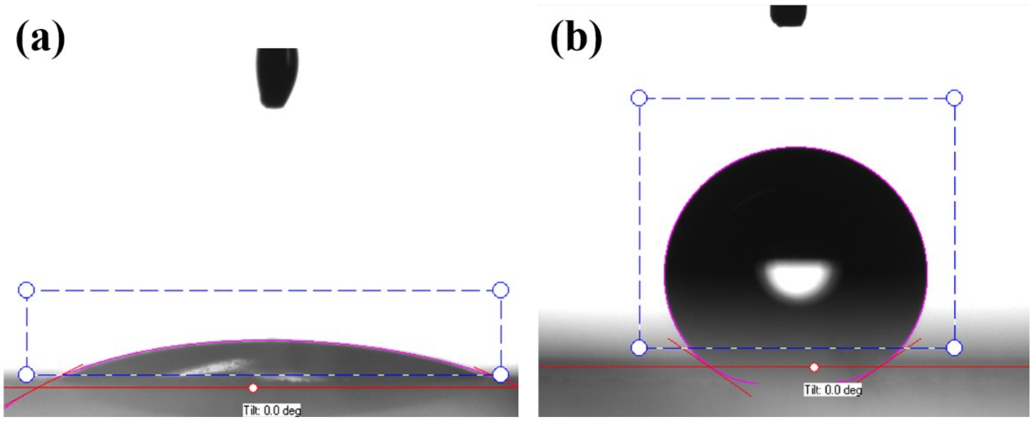

Fig. S4. Images of sessile drops on the surface of glass slides coated with (a) unmodified and (b) hydrophobic silane modified hollow silica particles. The fitting lines of the Young–Laplace equation to the surface of the sessile drops are also shown.

Young's modulus measurements on hollow silica particles:

Nanoindentation measurements were carried out on a Hysitron TriboIndenter. Measurements were performed on 50 different areas. The Young's modulus values of the hollow silica particles as a function of the maximum load (P_{max}) and maximum displacement (h_{max}) are shown in Figure S5. The average modulus value was 1.5 ± 0.5 GPa. The Young's modulus values of a silica glass typically range between 55 to 60 GPa [1]. Herein, the measured values are significantly lower due to the hollow structure of the silica particles.

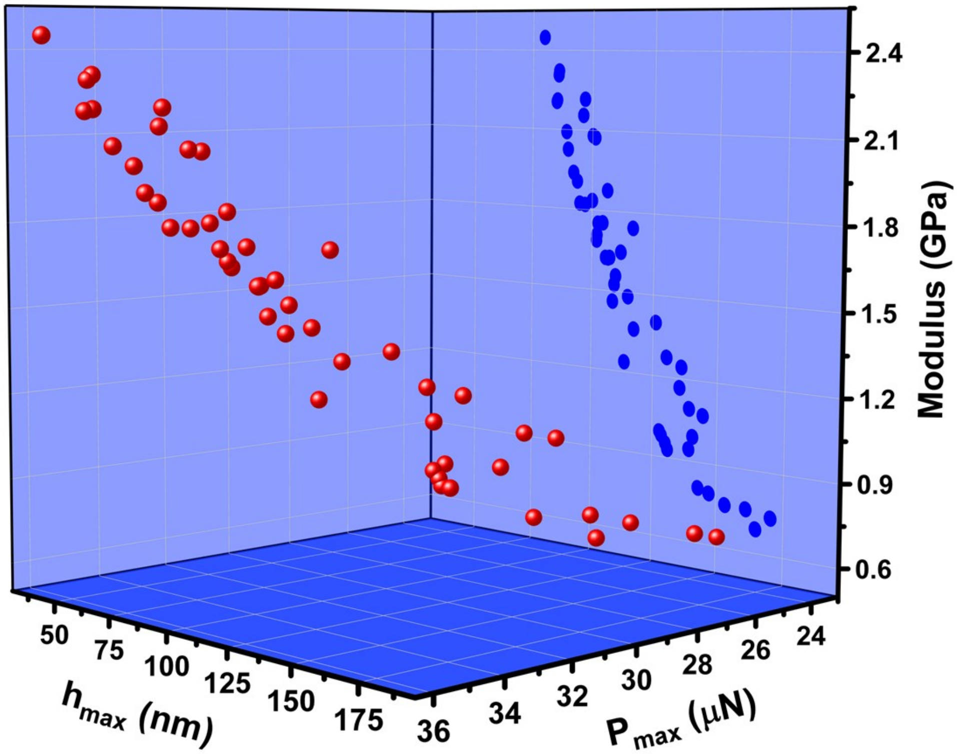

Fig. S5. Young's modulus values (read spheres) of the hollow silica particles as a function of the maximum load and maximum displacement. The projection of the modulus values on the YZ-plane are also shown (blue spheres).

Acknowledgement: We are thankful to 'Andres Marquez Rossy' for his help in Young's modulus measurements.

References

[1] J. Park, J. Sharma, M. Goswami, D. Voylov, G.G. Jang, M.G. Lassiter, A.M. Rossy and G. Polizos, *Sol. Energy Mater. Sol. Cells*, 2020, **206,** 110302.
